# Translation and validation of the STOP-Bang questionnaire into Slovene

**DOI:** 10.1186/s40001-021-00503-z

**Published:** 2021-04-07

**Authors:** Andrej Pangerc, Marija Petek Šter, Leja Dolenc Grošelj

**Affiliations:** 1 Community Health Centre Bled, Bled, Slovenia; 2 Community Health Centre Trebnje, Trebnje, Slovenia; 3 Department of Family Medicine, Faculty of Medicine, University of Ljubljana, Ljubljana, Slovenia; 4 Institute of Clinical Neurophysiology, Division of Neurology, University Medical Centre Ljubljana, Ljubljana, Slovenia; 5 Department of Neurology, Faculty of Medicine, University of Ljubljana, Ljubljana, Slovenia

**Keywords:** Apnoea–hypopnoea index, Obstructive sleep apnoea, Slovene translation, STOP-Bang questionnaire, Validation

## Abstract

**Purpose:**

To translate, culturally adapt and evaluate the Slovene version of the STOP-Bang questionnaire (SBQ) for use in the sleep clinic.

**Methods:**

Standard forward–backward translation and harmonisation of the Slovene translation of the SBQ were performed. Test–retest reliability was performed on a sample of healthy subjects. A cross-sectional study was performed with patients referred for a sleep study. Patients filled out the Slovene translation of the SBQ before undergoing sleep study.

**Results:**

The validation group consisted of 256 patients, of which 237 (92.6%) were included. Mean age was 52.5 ± 14.6, 63.3% of patients were male. Obstructive sleep apnoea (OSA) (apnoea–hypopnea index (AHI) ≥ 5) was present in 69.6% of patients, of whom 22.4% had mild (AHI ≥ 5 and < 15), 21.9% moderate (AHI ≥ 15 and < 30), and 25.3% severe (AHI ≥ 30) OSA. A SBQ score of 3 had a sensitivity of 92.1 (86.9–95.7), specificity of 44.4 (32.7–56.6), PPV of 79.2 (75.5–82.4) and AUC of 0.757 (95% CI 0.692–0.823; *p* < 0.001) for all OSA (AHI ≥ 5). Each increase in the SBQ score was associated with an increase in the probability of OSA.

**Conclusion:**

This study shows that the Slovene version of the SBQ is a valid tool for evaluating the risk of OSA in a sleep clinic.

**Supplementary Information:**

The online version contains supplementary material available at 10.1186/s40001-021-00503-z.

## Introduction

Obstructive sleep apnoea (OSA) is the most common sleep-related respiratory disorder which is recognized as an independent risk factor for a range of clinical conditions, such as hypertension, stroke, depression and diabetes [1, 2]. Moreover, OSA is a significant cause of motor vehicle crashes [3] and is associated with an increase in all-cause mortality, particularly due to coronary artery disease [3, 4]. It has been estimated that up to 80% of individuals with moderate-to-severe OSA have not been diagnosed [5].

Polysomnography as the current gold standard for OSA diagnosis is expensive and difficult to set up and interpret [1]. Portable home monitoring (type III polygraphy) was approved by the American Academy of Sleep Medicine as an alternative in patients without significant cardiorespiratory disease, chronic opioid medication use, history of stroke, or severe insomnia [6].

High prevalence of undiagnosed OSA, limited resources and the short- and long-term consequences of the disease have created a need to develop a reliable and affordable screening tool for OSA risk stratification. Questionnaires can be appropriate tools to that end since they can be applied and scored easily as part of routine daily practice [7]. The STOP-Bang questionnaire (SBQ) is a simple and validated questionnaire that detects OSA with high sensitivity and is, therefore, more suited for a sleep clinic setting compared other questionnaires as it helps to avoid missing cases [8, 9]. In their 2017 meta-analysis, Chiu et al. [10] compared the Berlin questionnaire, the SBQ and Epworth’s sleepiness scale in terms of OSA detection. The results revealed that for mild, moderate, and severe OSA, the pooled sensitivity and diagnostic odds ratio of the SBQ were significantly higher in comparison to the other screening questionnaires. The SBQ also demonstrated good flexibility as it had the largest area under the curve when compared to seven other questionnaires for the commonly used AHI cutoffs of 5, 15 and 30 [11]. The SBQ has been translated and validated in numerous languages, but no scientifically produced translation or validation of a Slovene version has, thus, far been produced. We aim to translate, culturally adapt and validate the SBQ for use with Slovene patients.

## Methods

We split our study into two parts: first, we translated and adapted the SBQ and tested its internal consistency; second, the translated SBQ was validated against sleep study in a cross-sectional study.

### Study population

From February to April 2017, a sample of 153 healthy Slovene-speaking subjects aged 18 or older were recruited for test–retest reliability. 134 (87.6%; mean age 42.9 ± 12.7) completed both sets of the SBQ required for final analysis. The demographic characteristics and SBQ results of this sample are shown in Table 1.

**Table 1:** Demographic characteristics and STOP-Bang output of the convenience sample volunteers

	*n* = 134
Age (years)	42.9 ± 12.7
Gender (male)	53 (40%)
STOP-Bang score	1.42 ± 1.25
Snoring	13 (9.6%)
Tiredness	42 (31.1%)
Observed apnoea	12 (8.9%)
Hypertension	13 (9.6%)
Age over 50	50 (37%)
Neck	5 (3.7%)
BMI (kg/m^2^)	24.71 (21.55–27.04)

The second part of the study was conducted at the sleep clinic at the Institute of Clinical Neurophysiology, University Medical Centre Ljubljana, Slovenia. All patients referred for polygraphy or polysomnography who were 18 or older and spoke Slovene were asked to participate in the study. Patients with neuromuscular conditions were excluded. There were no limitations of referrals. Of the 256 patients referred, 237 (92.6%; mean age 52.5 ± 14.6) were included in the final analysis. 16 patients failed to complete the questionnaires. Two patients were excluded for low fidelity polygraphy recordings which they were not willing to repeat. One was excluded, as he could not fall asleep with PG and declined further testing. The demographic characteristics of this second sample are shown in Table 2.

**Table 2:** Demographic characteristics of the patients recruited at the sleep clinic

	All *n* = 237
Age (years)	52.5 ± 14.6
Gender (male)	150 (63.3%)
Neck (cm), *n* = 207	41.42 ± 4.40
BMI (kg/m^2^)	29.69 (26.30–33.17)

## Study design and data collection

### Step 1: Translation of the SBQ

The SBQ was translated from English to Slovene by two independent researchers, one a medical doctor with experience in sleep medicine and the other a psychologist with experience in instrument development and translation. Both were native Slovene speakers proficient in English. A bilingual panel consisting of the two researchers who performed the forward translation and a medical doctor, a somnology specialist, conducted a synthesis of the two translations. An independent translator, psychologist by training, with no knowledge of the SBQ, who grew up in a Slovene–English bilingual home, conducted the back translation. To verify that the questions were understood correctly 10 adults (6 females and 4 males, mean age 39.3 ± 11.8) participated in a one-on-one think-aloud cognitive interview with a psychologist. Procedures were in line with the standards set out by the World Health Organisation [12].

### Step 2: Test–retest reliability of the SBQ

Participants were given two sets of questionnaires. The first set contained demographic questions, exclusion criteria and the SBQ. The second set consisted of the SBQ retest which was taken 2–3 weeks after the first.

### Step 3: Validation

#### Polysomnography and polygraphy

Patients referred to the sleep clinic for sleep study underwent either ambulatory type III polygraphy (PG) or type I polysomnography (PGS). PSG was used in patients with significant cardiorespiratory disease, chronic opioid medication use, history of stroke, or severe insomnia. When the PG recording could not be used due low fidelity or failure because of technical reasons such as the respiratory effort belt becoming loose, the dislodging of the pulse oximeter, etc. PSG was ultimately performed. This is in line with normal sleep centre operations and follows the recommendations by the American Association for Sleep Medicine (AASM) [6]. PG was recorded using the Alice NightOne, Phillips Respironics, system. PSG was recorded using the Alice 6, Phillips Respironics, system. Patients with neuromuscular conditions were excluded. This was done because other disorders such as sleep-onset insomnia, sleep maintenance insomnia, excessive eye movement sleep behaviour disorder, central sleep apnoeas, and diaphragm weakness with pseudo-central apnoeas, for which the SBQ was not designed, are common in this population [13].

Recordings were manually scored in our accredited sleep centre by our most experienced certified sleep specialist. Scoring was conducted in accordance with AASM guidelines and rules [6, 14]. The scorer was blinded to patients’ clinical histories and SBQ scores. The severity of OSA was determined as mild for apnoea hypopnea index (AHI) ≥ 5 and < 15, moderate for AHI ≥ 15 and < 30, and severe for AHI ≥ 30 [6, 14]. In the final analysis, datasets with complete STOP-Bang questionnaires and good-quality recordings were included.

### Statistical analysis

Patients’ characteristics were presented with the mean (standard deviation) in the case of normally distributed numerical variables; median (interquartile range) in the case of non-normally distributed numerical variables; and with frequencies (%) in the case of categorical variables. The differences between the OSA and non-OSA group were tested with independent *t* test or Mann–Whitney test, while the Chi-square test was used for categorical variables. The validation of the SBQ included the evaluation of internal consistency (Cronbach’s Alpha) and test–retest reliability (Gwet’s AC1 agreement coefficient). Reliability was also tested with factor analysis using tetrachoric correlations. To assess the predictive validity of the SBQ, sensitivity, specificity, positive predictive value (PPV) and negative predictive value (NPV) were calculated for different AHI cutoffs. Logistic regression was used to calculate the predicted probabilities for AHI scores.

## Results

### Translation and adaptation

After the forward translations were finished, they were examined by a bilingual panel and a consensus translation was reached. No major discrepancies were noted between the original English and the back-translated version. After reviewing the results of cognitive interviewing, the panel decided to break down the question whether the body mass index (BMI) was 35 or higher into its constituents, meaning that patients were instead asked to give their weight and height as this was easier to self-report. See Additional file 1 for the final version of the Slovene SBQ.

### Internal consistency and temporal stability

Cronbach’s Alpha coefficient for the 8 items was 0.628. Factor analysis suggested a single-component solution that explained 32.2% of the total item variance. The loadings for each of the SBQ items are presented in Table 3. The intraclass correlation coefficient between test and retest total scores was 0.94 (95% CI 0.91–0.95, *p* < 0.001). The test–retest reliability for each item was assessed with Gwet’s AC1 coefficients and almost all scores were greater than 0.9, indicating excellent test–retest reliability (Table 4).

**Table 3:** Factor loadings based on principal component analysis with oblimin rotation for the eight items from the SBQ (*n* = 372)

	Loading
Do you snore loudly…	0.46
Do you often feel tired…	0.25
Has anyone observed…	0.40
Do you have or…	0.58
Is your age greater…	0.20
Gender male…	0.63
Is your BMI…	0.62
Does your neck…	0.99

**Table 4:** Test–retest reliability

Item	Gwet AC1 (95% CI)
Do you snore loudly…	0.95 (0.90–0.99)
Do you often feel tired…	0.74 (0.63–0.86)
Has anyone observed…	0.96 (0.93–1.00)
Do you have or …	1
Is your age greater …	0.99 (0.96–1.00)
Gender male …	1
Is your BMI …	0.99 (0.98–1.00)
Does your neck …	0.99 (0.98–1.00)

### Validation against AHI

A comparison of the data for patients who had OSA (AHI ≥ 5) with those who did not reveals a significant difference in demographic characteristics between the two groups (Table 5).

**Table 5:** Demographic characteristics of the patients recruited at the sleep clinic and a comparison of the data for those with and without OSA

	All *n* = 237	No OSA (AHI < 5) *n* = 72	OSA (AHI ≥ 5) *n* = 165	*p* value
Age (years)	52.5 ± 14.6	45.5 ± 14.7	55.6 ± 13.5	< 0.001
Gender (male)	150 (63.3%)	32 (44.4%)	118 (71.5%)	< 0.001
Neck (cm), *n* = 207	41.42 ± 4.40	38.88 ± 3.80	42.60 ± 4.16	< 0.001
BMI (kg/m^2^)	29.69 (26.30–33.17)	26.63 (23.25–30.29)	30.68 (27.84–34.23)	< 0.001

**Fig. 1:**
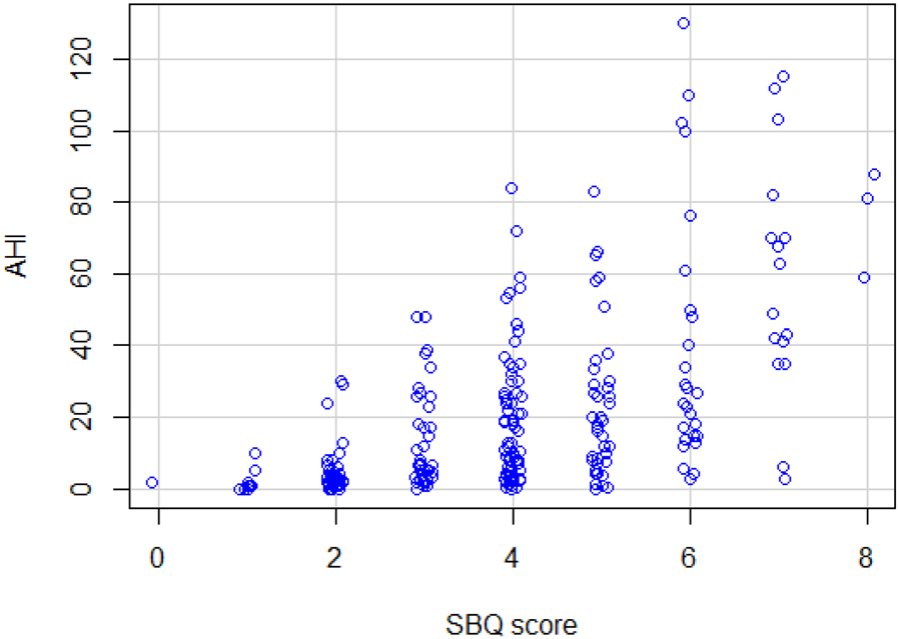
Scatter plot of total SBQ scores against AHI

**Fig. 2:**
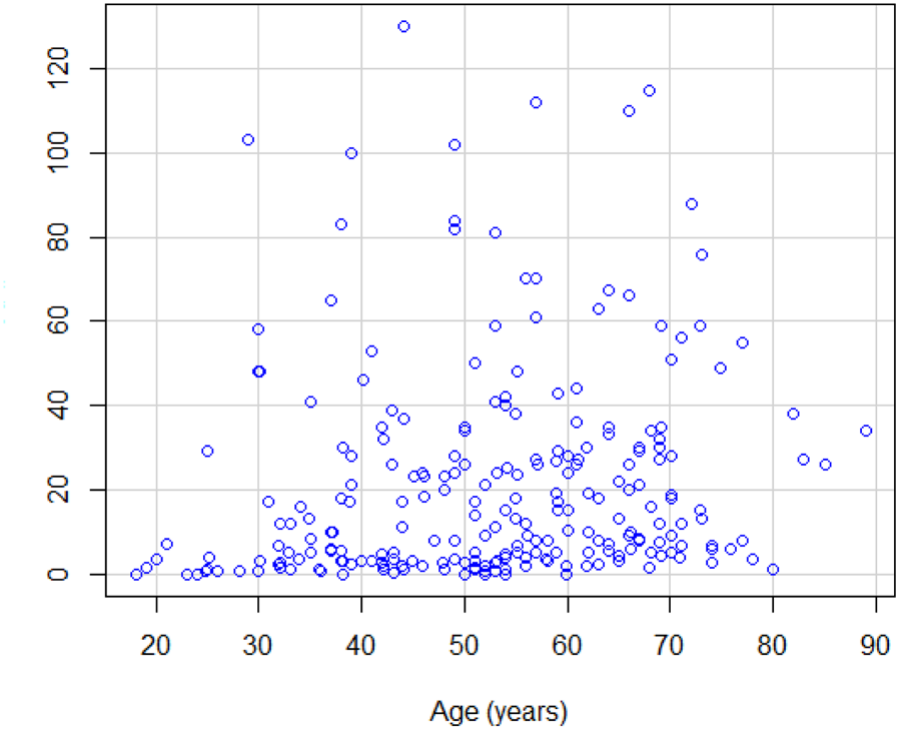
Scatter plot of age against AHI

**Fig. 3:**
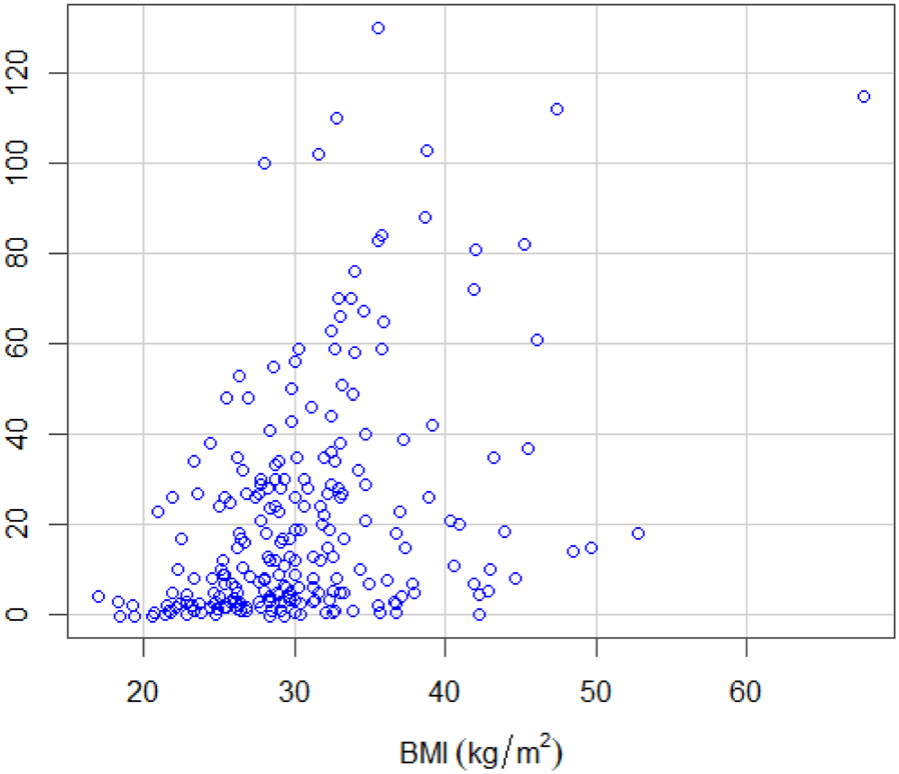
Scatter plot of BMI against AHI

**Fig. 4:**
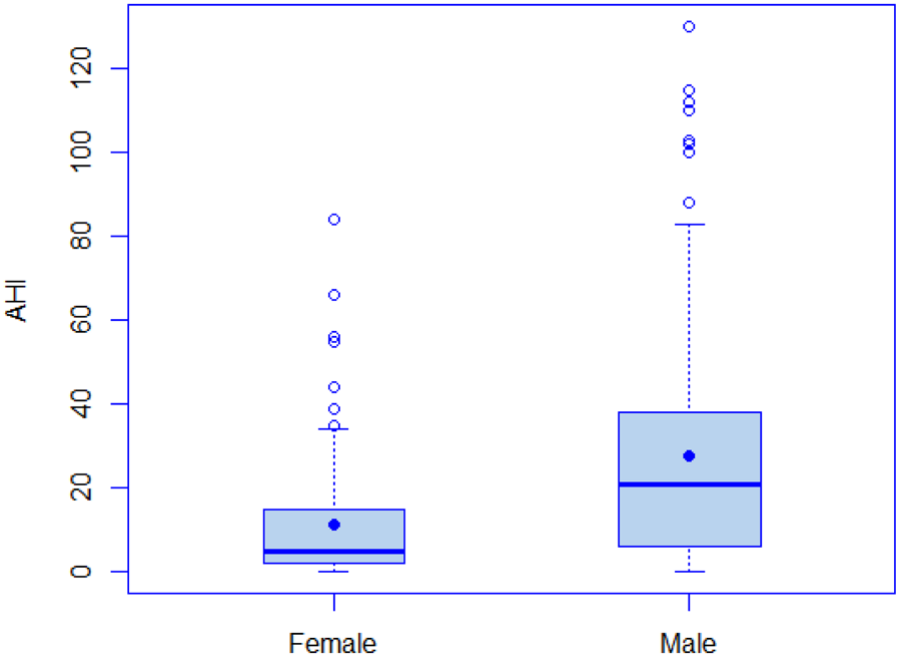
Box plot of sex against AHI

**Fig. 5:**
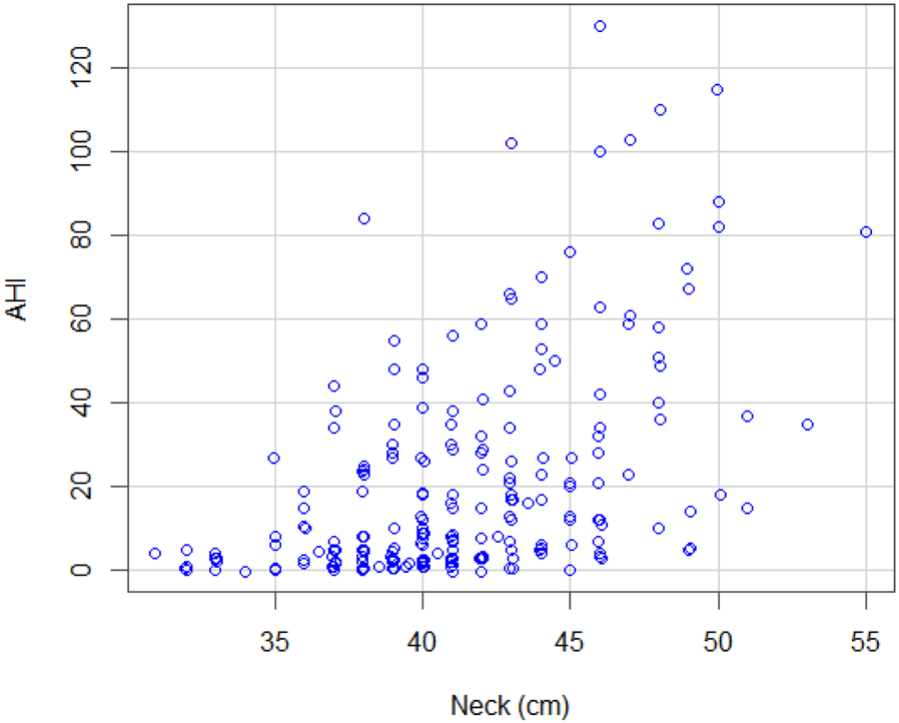
Scatter plot of neck circumference against AHI

**Fig. 6:**
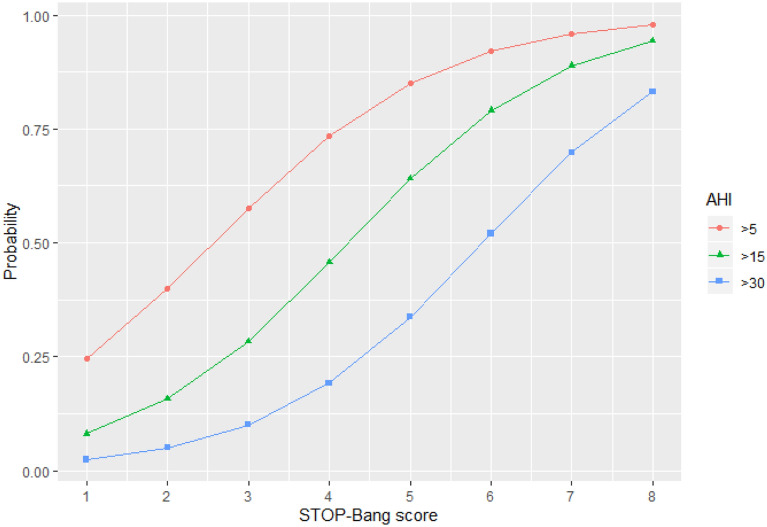
Predicted probabilities of having OSA of different severity based on the STOP-Bang questionnaire

Of the 237 patients at the sleep clinic, 72 patients (30.4%) had no OSA (AHI < 5), whereas the remaining 165 patients had mild (*n* = 53, 22.4%), moderate (*n* = 52, 21.9%) and severe OSA (*n* = 60, 25.3%). See Table 6 for comparison of the answers given to the SBQ and AHI between patients with and without OSA. As shown in Fig. 1, the total SBQ scores were positively associated with the AHI score. The correlation coefficient was 0.56 and statistically significant (95% CI 0.47–0.64; *p* < 0.001). 5 patients underwent PSG, while the rest had PG. The SBQ was evaluated with the cutoff values of AHI ≥ 5, 15 and 30; the respective areas under the ROC curve were 0.757 (95% CI 0.692–0.823; *p* < 0.001), 0.768 (95% CI 0.711–0.825; *p* < 0.001) and 0.77 (95% CI 0.704–0.836; *p* < 0.001). Plots for age, BMI, sex and neck circumference against AHI are also given for comparison in Figs. 2, 3, 4 and 5, respectively. As the SBQ score increased, sensitivities and NPV decreased whereas specificities and PPV increased. Detailed results are presented in Table 7. Predicted probabilities of having OSA based on the SBQ were calculated. As the SBQ increased from 3 to 8, the probabilities of OSA also increased (Fig. 6).

**Table 6:** Comparison of the answers to the STOP-Bang questionnaire and AHI in patients with and without OSA

	All *n* = 237	No OSA (AHI < 5) *n* = 72	OSA (AHI ≥ 5) *n* = 165	*p* value
Snoring	139 (58.6%)	32 (44.4%)	107 (64.8%)	0.003
Tiredness	176 (74.3%)	60 (83.3%)	116 (70.3%)	0.035
Observed apnoea	106 (44.7%)	20 (27.8%)	86 (52.1%)	0.001
Pressure	102 (43%)	21 (29.2%)	81 (49.1%)	0.004
BMI ≥ 35	44 (18.6%)	9 (12.5%)	35 (21.2%)	0.113
Age over 50 years	144 (60.8%)	32 (44.4%)	112 (67.9%)	0.001
Neck	101 (42.6%)	12 (16.7%)	89 (53.9%)	< 0.001
Gender (male)	150 (63.3%)	32 (44.4%)	118 (71.5%)	< 0.001
STOP-Bang score	4.06 ± 1.62	3.03 ± 1.42	4.51 ± 1.48	< 0.001
AHI	12.0 (3.5–30.0)	2.0 (0.77–3.0)	24.0 (10.75–39.5)	< 0.001

**Table 7:** Diagnostic ability of the SBQ scores from 1 to 8 at AHI cutoff points 5, 15 and 30 (*n* = 237)

SBQ score cutoff	*n* (%)	Sensitivity (95% CI)	Specificity (95% CI)	PPV (95% CI)	NPV (95% CI)
Any OSA (AHI ≥ 5)					
1	165 (69.6)	100.0 (97.8–100.0)	1.4 (0.04 –7.5)	69.9 (69.3–70.5)	100.0
2	163 (68.8)	98.8 (95.7–99.9)	11.1 (4.9–20.7)	71.8 (70.1–73.5)	80.0 (46.6–94.8)
3	152 (64.1)	92.1 (86.9–95.7)	44.4 (32.7–56.6)	79.2 (75.5–82.4)	71.1 (57.9–81.5)
4	125 (52.7)	75.8 (86.5–82.1)	61.1 (48.9–72.4)	81.7 (76.7–85.8)	52.4 (44.2–60.4)
5	75 (31.6)	45.5 (37.7–53.4)	84.7 (34.1–92.1)	87.2 (79.4–92.3)	40.4 (36.4–44.6)
6	43 (18.1)	36.1 (29.3–43.4)	93.5 (82.1–98.6)	95.8 (88.3–98.6)	26.1 (23.6–28.7)
7	18 (7.6)	10.9 (6.6–16.7)	98.6 (92.5–99.7)	94.7 (71.0–99.3)	32.6 (31.4–44.71)
8	3 (1.2)	1.8 (0.4–5.2)	100.0 (95.0–100.0)	100.0	30.8 (30.3–31.2)
Moderate and severe OSA (AHI ≥ 15)					
1	112 (47.3)	100.0 (96.8–100.0)	0.8 (0.02–4.4)	47.5 (47.1–47.9)	100.0
2	112 (47.3)	100.0 (96.8–100.0)	8.0 (3.9–14.22)	49.3 (48.1–50.6)	100.0
3	109 (46.0)	97.3 (92.4–99.4)	33.6 (25.4–42.6)	56.8 (53.6–59.9)	93.3 (81.7–97.8)
4	94 (39.7)	83.9 (75.8–90.2)	52.8 (43.7–61.8)	61.4 (56.6–66.1)	78.6 (70.0–85.2)
5	61 (25.8)	54.5 (44.8–63.9)	80.0 (71.9–86.6)	70.9 (62.3–78.3)	66.2 (61.1–71.0)
6	38 (16.0)	33.9 (25.3–43.5)	93.6 (87.8–97.2)	82.6 (69.8–90.7)	61.3 (57.9–64.5)
7	17 (7.2)	15.2 (9.1–23.2)	98.4 (94.3–99.8)	89.5 (66.8–97.3)	56.4 (54.4–58.4)
8	3 (1.2)	2.7 (0.6–7.6)	100.0 (97.1–100.0)	100.0	53.4 (52.7–54.2)
Severe OSA (AHI ≥ 30)					
1	60 (25.3)	100.0 (94.0–100.0)	0.6 (0.01–3.1)	25.4 (25.2–25.6)	100.0
2	60 (25.3)	100.0 (94.0–100.0)	5.6 (2.7–10.1)	26.4 (25.7–27.1)	100.0
3	59 (24.9)	98.3 (91.1–99.9)	24.9 (18.7–31.9)	30.7 (28.8–32.7)	97.8 (86.1–99.7)
4	54 (22.8)	90.0 (79.5–96.2)	44.1 (36.6–51.7)	35.3 (31.8–38.9)	92.9 (85.7–96.6)
5	37 (15.6)	61.7 (48.2–73.9)	72.3 (65.1–78.8)	43.0 (35.6–50.7)	84.8 (80.0–88.6)
6	27 (11.4)	45.0 (32.1–58.4)	89.3 (83.8–93.4)	58.7 (46.1–70.3)	82.7 (79.1–85.8)
7	17 (7.2)	28.3 (17.5–41.4)	98.9 (96.0–99.9)	89.5 (66.9–97.3)	80.3 (77.6–82.7)
8	3 (1.2)	5.0 (1.0–13.9)	100.0 (97.9–100.0)	100.0	76.0 (70.0–81.3)

## Discussion

During the process of cognitive interviewing, it was suggested that the question of BMI be broken down into its components, body mass and height, which would make it easier to use and understand. We chose to adopt this suggestion. We were unable to find any other validation studies of the SBQ where this was done.

Our translation of the STOP-Bang questionnaire showed good temporal stability. Intraclass correlation coefficient between test and retest total scores was high.

Internal consistency was evaluated with two statistical methods. The first was calculating Cronbach’s alpha, which was somewhat low at 0.63. This on par with 0.62 for the Brazilian translation [15], close to 0.7 for the Arab translation [16] and higher than the Lithuanian translations’ 0.41 [17]. We also performed factor analysis, which is more suitable for dichotomous variables, [18, 19] such as are found in the SBQ. This showed good internal consistency for six out of the questionnaire’s eight items. Item number two “Do you often feel tired, fatigued or sleepy during the daytime?” and item six “Age older than 50?” had a loading score below the threshold of 0.3. For item two, this could be explained by other common causes of tiredness other than OSA, for instance depression, which are common in the general population. Another cause could be the fact that some patients with OSA do not experience excessive daytime sleepiness [20]. The low factor loading of item five that refers to age could perhaps be explained by the fact that the study population consisted mostly of middle-aged subjects and a larger sample could perhaps have been more telling. Dr. Chung, who designed the questionnaire, did not evaluate internal consistency, citing that the questionnaire reflected four different dimensions of OSA morbidity and that internal consistency checking was, thus, not applicable [21]. Internal consistency checking was nevertheless performed in certain validation studies [7, 16] and omitted in others [17, 22, 23]. When it was carried out, Cronbach’s coefficient alpha was used, and values were typically low.

The prevalence of OSA (AHI of ≥ 5) in our sleep clinic population was 69.6%. For the Portuguese version, the prevalence was 78% [7], for the Lithuanian, this was 93% [17], for the Arabic, it was 94% [16] and for the Malayan, it was 100% [23].

A comparison of the answers given by patients with and without OSA (AHI ≥ 5) showed significant differences in all but one of the eight questions, i.e. the question referring to a BMI ≥ 35 where the *p* value was 0.113. This was somewhat surprising considering that OSA has been reported in over 40% of persons with a BMI of more than 30 [20]. Nevertheless, of the 44 patients with a BMI ≥ 35, only 9 did not have OSA. Interestingly, Reis et al. [7] also found BMI to be statistically nonsignificant. The specific cutoff value used for the BMI might be the cause. This sentiment is supported by Fig. 3 and Table 5 which show a correlation between the BMI and AHI.

For a SBQ score of 3, we found that the area under the ROC curve was high at 0.757 (95% CI 0.692–0.823; *p* < 0.001) for all OSA (AHI ≥ 5). This increased slightly for moderate/severe and severe OSA to 0.768 (95% CI 0.711–0.825; *p* < 0.001) and 0.77 (95% CI 0.704–0.836; *p* < 0.001), respectively. The AUC for all OSA (AHI ≥ 5) obtained by Reis et al. [7] was slightly higher than ours at 0.806 (95% CI 0.730–0.881), but slightly lower for moderate/severe (AHI ≥ 15) at 0.730 (95% CI 0.661–0.798) and severe OSA at 0.728 (0.655–0.801).

Among patients referred to the sleep clinic the Slovenian version of SBQ, at a score of 3, showed a high sensitivity 92.1% (86.9–95.7%) and moderate specificity of 44.4% (32.7–56.6%) for all OSA (AHI ≥ 5). This was on par with benchmarks such as Chung et al. [21], who had a sensitivity of 72.1% and specificity of 38.2% and Silva et al. [8] with sensitivity of 82.0% and specificity of 43.3% for the same range and cutoff. Low specificity was also observed in a number of translations [7, 16, 17] as well as meta-analysis by Nagappa et al. [9].

The PPV for an SBQ score of 3 for any OSA (AHI ≥ 5) was high at 79.2 (75.5–82.4). Specificity and PPV increased continuously for every increase in the SBQ. These results were on par with other translations of the SBQ [7, 24]. High sensitivity and PPV are essential for screening tools, but it could be argued that NPV is perhaps even more important for risk stratification. Our results show that a STOP-Bang score of 2 had a NPV of 80.0% (46.6–94.8) for all OSA (AHI ≥ 5) and 100.0% for moderate/severe (AHI ≥ 15) and severe OSA (AHI ≥ 30). Although our NPV might be higher due to an underestimation of the AHI brought about by the high percentage of sleep studies conducted with PG, the results are similar those obtained by Portuguese researchers [7].

A SBQ of 3 was chosen as the recommended cutoff. This is in line with other recent translations [7, 16, 17, 23].

An important study limitation was that the population referred to the sleep clinic was in a sense already pre-screened by referring practitioners. Our findings, thus, cannot be extended to other settings.

In our study, we primarily utilized PG, which accounted for 97.8% of all recordings. PG devices do not include sleep staging and can give lower AHI compared with PSG where periods of wakefulness are excluded from the calculation of AHI [25]. PG has, however, been shown to be a reliable alternative to PSG and is becoming ever more prevalent in clinical practice [7, 26]. Ours was not the first study to have used PG for validation of the STOP-Bang questionnaire [7].

## Conclusion

Our study has shown that the Slovene version of the SBQ is a simple, reliable and valid tool for the stratification OSA risk among Slovenes referred to a sleep clinic with high sensitivity and moderate specificity.

## Supplementary Information

**Additional file 1:** **Appendix 1**. Slovene translation of the STOP-Bang questionnaire

## Data Availability

Data and material are available upon request.

## Code availability

Sleep scoring was done using AASM visual scoring guidelines Sleepware G3 version 3.9.1 by Respironics inc. PA, USA. Statistical calculations and statistical graphic representations were produced using R Core Team (2019). R: A language and environment for statistical computing. R Foundation for Statistical Computing, Vienna, Austria.

## References

[1] Eastwood PR, Malhotra A, Palmer LJ, Kezirian EJ, Horner RL, Ip MS, Thurnheer R, Antic NA, Hillman DR (2010). Obstructive sleep apnoea: from pathogenesis to treatment: current controversies and future directions. Respirology.

[2] Neves Junior JAS, Fernandes APA, Tardelli MA, Yamashita AM, Moura S, Tufik S, Silva H (2020). Cutoff points in STOP-Bang questionnaire for obstructive sleep apnea. Arq Neuropsiquiatr.

[3] Kendzerska T, Gershon AS, Hawker G, Leung RS, Tomlinson G (2014). Obstructive sleep apnea and risk of cardiovascular events and all-cause mortality: a decade-long historical cohort study. PLoS Med.

[4] Punjabi NM, Caffo BS, Goodwin JL, Gottlieb DJ, Newman AB, O'Connor GT, Rapoport DM, Redline S, Resnick HE, Robbins JA, Shahar E, Unruh ML, Samet JM (2009). Sleep-disordered breathing and mortality: a prospective cohort study. PLoS Med.

[5] Young T, Evans L, Finn L, Palta M (1997). Estimation of the clinically diagnosed proportion of sleep apnea syndrome in middle-aged men and women. Sleep.

[6] Kapur VK, Auckley DH, Chowdhuri S, Kuhlmann DC, Mehra R, Ramar K, Harrod CG (2017). Clinical practice guideline for diagnostic testing for adult obstructive sleep apnea: an American academy of sleep medicine clinical practice guideline. J Clin Sleep Med.

[7] Reis R, Teixeira F, Martins V, Sousa L, Batata L, Santos C, Moutinho J (2015). Validation of a Portuguese version of the STOP-Bang questionnaire as a screening tool for obstructive sleep apnea: analysis in a sleep clinic. Rev Port Pneumol.

[8] Silva GE, Vana KD, Goodwin JL, Sherrill DL, Quan SF (2011). Identification of patients with sleep disordered breathing: comparing the four-variable screening tool, STOP, STOP-Bang, and Epworth sleepiness scales. J Clin Sleep Med.

[9] Nagappa M, Liao P, Wong J, Auckley D, Ramachandran SK, Memtsoudis S, Mokhlesi B, Chung F (2015). Validation of the STOP-Bang questionnaire as a screening tool for obstructive sleep apnea among different populations: a systematic review and meta-analysis. PLoS ONE.

[10] Chiu HY, Chen PY, Chuang LP, Chen NH, Tu YK, Hsieh YJ, Wang YC, Guilleminault C (2017). Diagnostic accuracy of the Berlin questionnaire, STOP-Bang, STOP, and Epworth sleepiness scale in detecting obstructive sleep apnea: a bivariate meta-analysis. Sleep Med Rev.

[11] Wang W, Yuan S, Le Grange JM, Zheng H, Yao T, Peng W, Zhang J (2020). Evaluating the performance of five scoring systems for prescreening obstructive sleep apnea–hypopnea syndrome. Sleep Breath.

[12] WHO. Process of translation and adaptation of instruments. World Health Organization. 2007. https://www.who.int/substance_abuse/research_tools/translation/en/. Accessed 7 June 2020.

[13] Gaig C, Iranzo A (2012). Sleep-disordered breathing in neurodegenerative diseases. Curr Neurol Neurosci Rep.

[14] Berry RB, Budhiraja R, Gottlieb DJ, Gozal D, Iber C, Kapur VK, Marcus CL, Mehra R, Parthasarathy S, Quan SF, Redline S, Strohl KP, Davidson Ward SL, Tangredi MM (2012). Rules for scoring respiratory events in sleep: update of the 2007 AASM manual for the scoring of sleep and associated events. Deliberations of the sleep apnea definitions task force of the American Academy of Sleep Medicine. J Clin Sleep Med.

[15] Fonseca LB, Silveira EA, Lima NM, Rabahi MF (2016). STOP-Bang questionnaire: translation to Portuguese and cross-cultural adaptation for use in Brazil. J Bras Pneumol.

[16] BaHammam AS, Al-Aqeel AM, Alhedyani AA, Al-Obaid GI, Al-Owais MM, Olaish AH (2015). The validity and reliability of an arabic version of the STOP-Bang questionnaire for identifying obstructive sleep apnea. Open Respir Med J.

[17] Balsevičius T, Vaitukaitienė G, Šaduikytė B, Miliauskas S, Pribuišienė R (2021). Validating the Lithuanian version of the STOP-BANG questionnaire for diagnosing obstructive sleep apnea. Sleep Breath.

[18] Knol DL, Berger MP (1991). Empirical comparison between factor analysis and multidimensional item response models. Multivar Behav Res.

[19] Lorenzo-Seva U, Ferrando PJ (2012). TETRA-COM: a comprehensive SPSS program for estimating the tetrachoric correlation. Behav Res Methods.

[20] Veasey SC, Rosen IM (2019). Obstructive sleep apnea in adults. N Engl J Med.

[21] Chung F, Yegneswaran B, Liao P, Chung SA, Vairavanathan S, Islam S, Khajehdehi A, Shapiro CM (2008). STOP questionnaire: a tool to screen patients for obstructive sleep apnea. Anesthesiology.

[22] Bille J, Bille-Hasselstrom C, Petersen CG (2015). Translation and validation of the STOP-Bang questionnaire for obstructive sleep apnoea into Danish. Dan Med J.

[23] Abdullah B, Idris AI, Mohammad ZW, Mohamad H (2018). Validation of Bahasa Malaysia STOP-BANG questionnaire for identification of obstructive sleep apnea. Sleep Breath.

[24] Duarte RLM, Fonseca LBM, Magalhães-da-Silveira FJ, Silveira EAD, Rabahi MF (2017). Validation of the STOP-Bang questionnaire as a means of screening for obstructive sleep apnea in adults in Brazil. J Bras Pneumol.

[25] Randerath W, Bassetti CL, Bonsignore MR, Farre R, Ferini-Strambi L, Grote L, Hedner J, Kohler M, Martinez-Garcia MA, Mihaicuta S, Montserrat J, Pepin JL, Pevernagie D, Pizza F, Polo O, Riha R, Ryan S, Verbraecken J, McNicholas WT (2018). Challenges and perspectives in obstructive sleep apnoea: report by an ad hoc working group of the Sleep Disordered Breathing Group of the European Respiratory Society and the European Sleep Research Society. Eur Respir J.

[26] El Shayeb M, Topfer LA, Stafinski T, Pawluk L, Menon D (2014). Diagnostic accuracy of level 3 portable sleep tests versus level 1 polysomnography for sleep-disordered breathing: a systematic review and meta-analysis. CMAJ.

