# Supplementary material for: Translation and validation of the STOP-Bang questionnaire into Slovene
Source: Eur J Med Res. 2021 Apr 7;26:32. doi: 10.1186/s40001-021-00503-z (PMC8025340; doi:10.1186/s40001-021-00503-z)
Supplement: Supplementary file 1 — Additional file 1: Appendix 1. Slovene translation of the STOP-Bang questionnaire [file 40001_2021_503_MOESM1_ESM.docx]

**Appendix 1** Slovene translation of the STOP-Bang questionnaire

Vprašalnik STOP-Bang za obstruktivno apnejo v spanju

|  | |  |
| --- | --- | --- |
| DA | NE | Ali glasno smrčite (glasneje od običajnega govora oziroma tako glasno, da vas slišijo skozi zaprta vrata)? |
|  |  |  |
| DA | NE | Ali se podnevi pogosto počutite utrujeni, izčrpani ali zaspani? |
|  |  |  |
| DA | NE | Ali je kdo opazil, da med spanjem prenehate dihati? |
|  |  |  |
| DA | NE | Ali imate visok krvni tlak oziroma se zdravite zaradi visokega krvnega tlaka? |
|  |  |  |
| Koliko tehtate? ____________kg | | |
|  |  | ITM* ≥ 35 DA NE |
| Kako visoki ste? ____________cm | | |
|  |  |  |
| DA | NE | Ste stari 50 let ali več? |
|  |  |  |
| DA | NE | Za ženske: Je obseg vašega vratu večji od 41 centimetrov?  Za moške: Je obseg vašega vratu večji od 43 centimetrov? |
|  |  |  |
| DA | NE | Ste moški? |
|  |  |  |
| ------------------------------------------------------------------------------------- | | |
|  |  |  |
| *ITM (indeks telesne mase) = $\frac{teža (kg)}{(višina {(m))}^{2}}$  Interpretacija: seštevek odgovorov DA enak ali večji od 3 predstavlja povišano tveganje za obstruktivno apnejo v spanju. | | |
